# Functional regulation of von Willebrand factor ameliorates acute ischemia-reperfusion kidney injury in mice

**DOI:** 10.1038/s41598-019-51013-2

**Published:** 2019-10-08

**Authors:** Shiro Ono, Hideto Matsui, Masashi Noda, Shogo Kasuda, Noritaka Yada, Kiyomi Yoshimoto, Masashi Akiyama, Toshiyuki Miyata, Mitsuhiko Sugimoto, Kenji Nishio

**Affiliations:** 1 Departments of General Medicine, Nara Medical University, Kashihara, Japan; 2 Departments of Legal Medicine, Nara Medical University, Kashihara, Japan; 3 Departments of Molecular Pathogenesis, National Cerebral and Cardiovascular Center, Suita, Japan; 4 Departments of Cerebrovascular Medicine, National Cerebral and Cardiovascular Center, Suita, Japan

**Keywords:** Biochemistry, Physiology

## Abstract

Acute kidney injury (AKI), an abrupt loss of renal function, is often seen in clinical settings and may become fatal. In addition to its hemostatic functions, von Willebrand factor (VWF) is known to play a role in cross-talk between inflammation and thrombosis. We hypothesized that VWF may be involved in the pathophysiology of AKI, major causes of which include insufficient renal circulation or inflammatory cell infiltration in the kidney. To test this hypothesis, we studied the role of VWF in AKI using a mouse model of acute ischemia-reperfusion (I/R) kidney injury. We analyzed renal function and blood flow in VWF-gene deleted (knock-out; KO) mice. The functional regulation of VWF by ADAMTS13 or a function-blocking anti-VWF antibody was also evaluated in this pathological condition. Greater renal blood flow and lower serum creatinine were observed after reperfusion in VWF-KO mice compared with wild-type (WT) mice. Histological analysis also revealed a significantly lower degree of tubular damage and neutrophil infiltration in kidney tissues of VWF-KO mice. Both human recombinant ADAMTS13 and a function-blocking anti-VWF antibody significantly improved renal blood flow, renal function and histological findings in WT mice. Our results indicate that VWF plays a role in the pathogenesis of AKI. Proper functional regulation of VWF may improve the microcirculation and vessel function in the kidney, suggesting a novel therapeutic option against AKI.

## Introduction

Acute kidney injury (AKI), defined as sudden renal failure or damage that occurs within a few hours or days, is often seen in clinical settings and its mortality remains high even in developed countries^1,2^. Ischemia-reperfusion (I/R) kidney injury, which may occur in various pathological conditions such as, shock, drug-induced renal ischemia, surgical procedures or transplantation, is a major potential cause of AKI^1–4^. However, the precise mechanisms underlying acute I/R injury are complex and not yet fully understood. Ischemia-induced endothelial dysfunction or excessive inflammatory responses may be involved in the pathophysiology of I/R injury, leading to acute organ failure^3–5^.

The adhesive protein von Willebrand factor (VWF) plays an essential role on hemostasis, mediating platelet adhesion and aggregation under rheological conditions with high shear stress^6–8^. Conversely, excessive VWF activity may trigger thrombotic complications. To prevent this *in vivo*, the metalloprotease ADAMTS13 precisely regulates VWF functions by reducing the VWF multimer size, which is the most relevant property of VWF in terms of its biological activity^9–11^. Recent mouse model studies demonstrated that VWF plays a role in the pathophysiology of various I/R injuries, such as myocardial infarction, brain stroke and liver injury^12–15^.

Accordingly, we hypothesized that VWF-dependent thrombotic or inflammatory responses may also be involved in I/R kidney injury. In this regard, a very recent study by others^16^ suggested that recombinant ADAMTS13 could be effective to improve renal ischemia-reperfusion injury. Using a mouse model of I/R kidney injury, we therefore explored the functional relevance of the VWF-ADAMTS13 axis in this pathologic condition and the therapeutic potential of regulating VWF functions.

## Results

### Effects of gene deletion of VWF or rADAMTS13 on RBF and serum creatinine value in mice with I/R kidney injury

**Figure 1:**
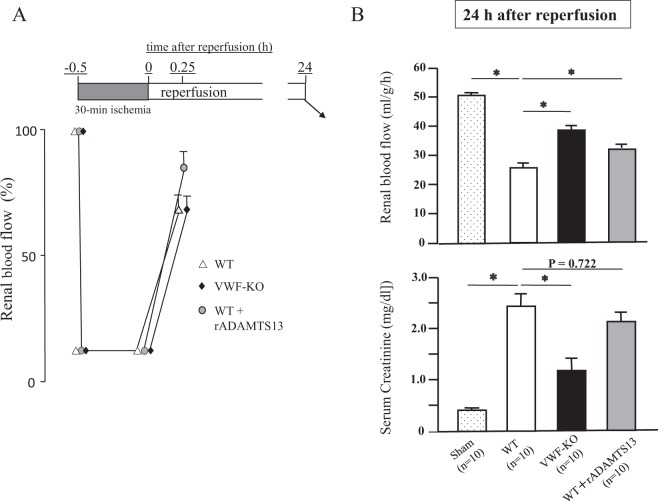
Effects of gene deletion of VWF or rADAMTS13 on RBF and serum creatinine in mice with I/R kidney injury. I/R injury was induced in the left kidneys of WT (n = 10) and VWF-KO mice (n = 10) (see the upper horizontal bar representing the I/R protocol); the right kidneys had been surgically removed 1 week prior to the I/R experiment. In another group of WT mice (n = 10), rADAMTS13 (10 μg/mouse) was injected intravenously 15 min before the I/R operation. (**A**) RBF, measured at 15 min after reperfusion by Laser Doppler flowmetry, is expressed as the percentage of the pre-ischemic value (mean ± SEM). During the ischemic phase, RBF is maintained at around 10% of the pre-ischemic value for 30 min. Then, the RBF in all mice tested are restored to approximately 65–85% of the pre-ischemic value at 15 min after reperfusion. (**B**) Upper panel; Bars indicate the mean actual RBF units ± SEM (ml/g/h, n = 10, each group; plus Sham operation as a negative control, n = 10) at 24 h after reperfusion. The RBF in VWF-KO mice is significantly (*p < 0.05) greater than that in WT mice 24 h after reperfusion. Note also that rADAMTS13 injection significantly (*p < 0.05) improved RBF in WT mice at 24 h after reperfusion. Lower panel; Bars indicate the mean ± SEM of serum creatinine values at 24 h after reperfusion. Consistent with the RBF data, the serum creatinine value in VWF-KO mice is significantly (*p < 0.05) lower than that in WT mice. The RBF and serum creatinine values demonstrated that kidney damages during reperfusion was less extensive in VWF-KO mice than in WT controls, and that the administration of rADAMTS13 significantly improved kidney damages in WT mice.

Experimental renal I/R was induced in WT mice and VWF-KO mice. In another group of WT mice, rADAMTS13 was administered 15 min before the I/R operation. During the ischemic phase, the RBF was maintained at around 10% of the pre-ischemic value for 30 min (Fig. 1A). Then, we confirmed that the RBF in all mice tested are restored to approximately 65–85% of the pre-ischemic value at 15 min after reperfusion (Fig. 1A). At 24 h after reperfusion, the RBF in VWF-KO mice (mean actual flow units ± SEM; 38.0 ± 2.9 ml/g/h) was significantly greater than that in WT mice (25.8 ± 2.7 ml/g/h, Fig. 1B), while both RBF values were almost the same at 15 min after reperfusion (WT; 69.2 ± 5.6%; VWF-KO mice; 68.1 ± 5.1% of the pre-ischemic value; Fig. 1A). The RBF in WT mice that received rADAMTS13 injection (32.2 ± 1.7 ml/g/h) was also significantly greater than that in WT mice at 24 h after reperfusion (Fig. 1B).

The mice were sacrificed for blood collection and histological analysis of kidney tissue 24 h after reperfusion. The serum creatinine value in VWF-KO mice at 24 h after reperfusion was significantly lower than that in WT mice (VWF-KO, 1.22 ± 0.25 mg/dl; WT, 2.41 ± 0.29 mg/dl; Fig. 1B). The RBF and serum creatinine values demonstrated that kidney damages during reperfusion was less extensive in VWF-KO mice than in WT controls, and that the administration of rADAMTS13 significantly improved kidney damages in WT mice.

### Histological analysis of kidney tissue from WT mice with or without rADAMTS13 infusion and fromVWF-KO mice after I/R kidney injury

**Figure 2:**
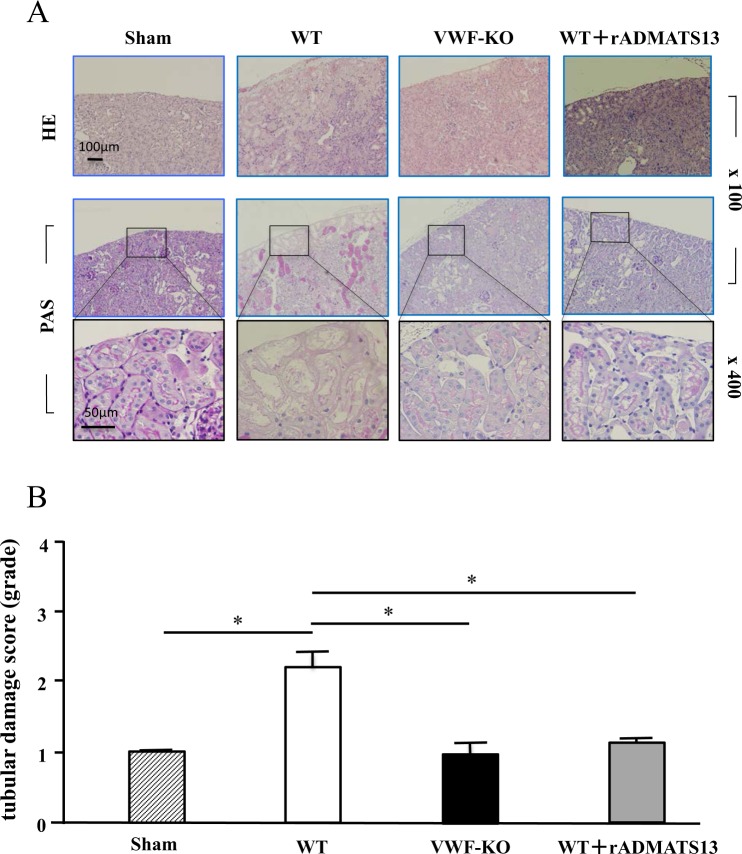
Histological analysis with HE and PAS staining of kidney tissue in WT mice with or without rADAMTS13 infusion and in VWF-KO mice. At 24 h after reperfusion (see the I/R protocol in Fig. 1), mice were sacrificed for histological analysis of kidney tissue. (**A**) Images of kidney tissue with HE staining (upper panels, original magnification, 100 ×) or PAS staining (middle panels, original magnification, 100 ×; and lower panels, enlarged images of areas enclosed in rectangles in corresponding middle panels, original magnification, 400 ×) are each representative of samples from 5 different mice. Note that tubular necrosis, as demonstrated by the loss of normal tubular construction (HE) or elimination of brush borders (PAS), is less severe in VWF-KO mice than WT mice, and rADAMTS13 injection ameliorated this tubular damage in WT mice. (**B**) Statistical analysis of the tubular damage scores corresponding to the above images; each bar represents the average (±SEM) of 15 areas (each 1 mm^2^; 3 areas were randomly selected from 5 individual mouse samples). Note that tubular damage is significantly (*) less severe in VWF-KO mice than in WT mice. Note also that rADAMTS13 injection significantly (*) improves tubular damage in WT mouse kidneys.

WT mice, in which most proximal tubules were damaged, demonstrated marked renal tubular necrosis just inside the renal capsules, characterized by the loss of normal tubular construction (upper panels in Fig. 2A, HE samples) or by the elimination of brush borders (equivalent to PAS-negative tubules in the middle and lower panels of Fig. 2A, PAS samples). This tubular necrosis was less severe in VWF-KO mice, in which the tubular architecture was largely preserved (Fig. 2A). Consistent with the renal blood flow data (see Fig. 1), rADAMTS13 injection ameliorated the kidney damage in WT mice (Fig. 2A). Statistical analysis using the tubular damage score (Fig. 2B) confirmed that kidney injury was significantly less severe in VWF-KO mice (1.07 ± 0.18) than in WT mice (2.17 ± 0.25). In addition, the score for WT mice in which rADAMTS13 was injected (1.15 ± 0.08) indicated significantly less tubular damage compared with WT mice that did not receive an rADAMTS13 injection (Fig. 2B).

**Figure 3:**
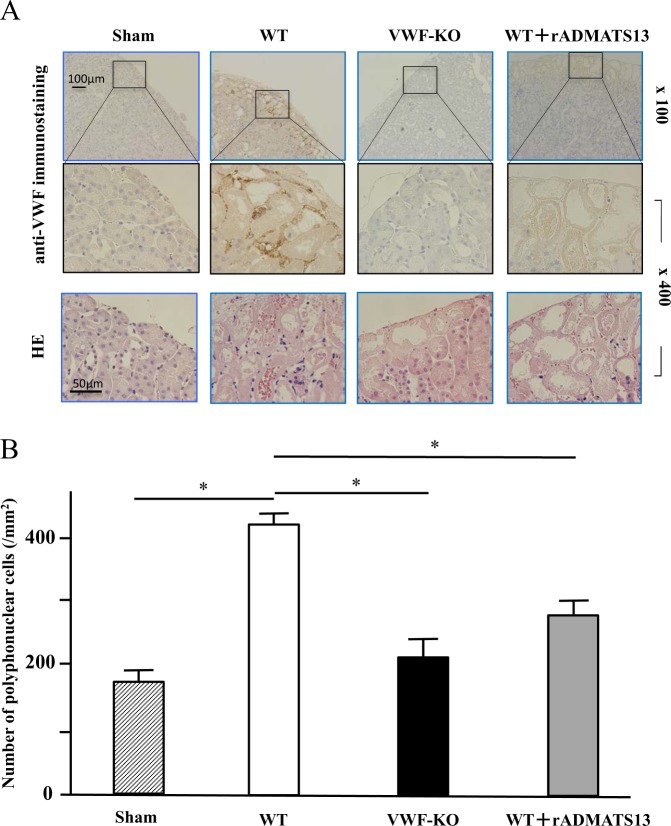
Histological analysis via anti-VWF immunostaining of kidney tissue in WT mice with or without rADAMTS13 infusion and in VWF-KO mice. Kidney tissues (the same as those described in the Fig. 2 legend) were subjected to immunostaining with anti-VWF antibody. (**A**) Images of kidney tissue following anti-VWF immunostaining (upper panels, original magnification, 100 ×; middle panels, enlarged images of areas enclosed in rectangles in corresponding upper panels, original magnification, 400 ×) or HE staining (lower panels, original magnification, 400 ×) are each representative of 5 independent mouse samples. Note that there is positive staining for VWF in the areas near or adjacent to necrotic tubules in WT mice, but no positive staining for VWF in VWF-KO-mice. Hardly any positive staining is seen in WT mice following ADAMTS13 injection. Like VWF staining, neutrophil infiltration around necrotic lesions is reduced in VWF-KO mice and following rADAMTS13 injection in WT mice (HE). (**B**) Statistical analyses of neutrophil accumulation corresponding to the above images; each bar represents the average (±SEM) of 15 areas (each 1 mm^2^; 3 areas were randomly selected from each of 5 individual mouse tissue samples with HE staining). Note that neutrophil accumulation within kidney tissues is significantly (*) decreased in VWF-KO mice. Note also that rADAMTS13 injection suppresses neutrophil infiltration in WT mouse kidney. These findings suggest close relationships between VWF and both tissue neutrophil infiltration and renal tubular damage.

Immunostaining of kidney tissues with anti-VWF antibody demonstrated prominent VWF staining near or adjacent to necrotic tubules in WT mice, while no positive-staining for VWF was observed in VWF-KO mice (Fig. 3A). Interestingly, hardly any positive staining was seen in WT mice when ADAMTS13 was injected (Fig. 3A). Consistent with the VWF distribution in kidney tissues, infiltration of polymorphonuclear cells that correspond to neutrophils was increased in WT mice compared with VWF-KO mice (HE-staining; Fig. 3A). Indeed, statistical analysis (Fig. 3B) confirmed that the neutrophil counts in kidney tissue samples from WT mice (413.1 ± 32.0/mm^3^) were significantly higher than those in samples from VWF-KO mice (209.2 ± 50.0/mm^3^).

### Effects of the function-blocking anti-VWF antibody NMC-4 on RBF and serum creatinine value in mice with I/R kidney injury

**Figure 4:**
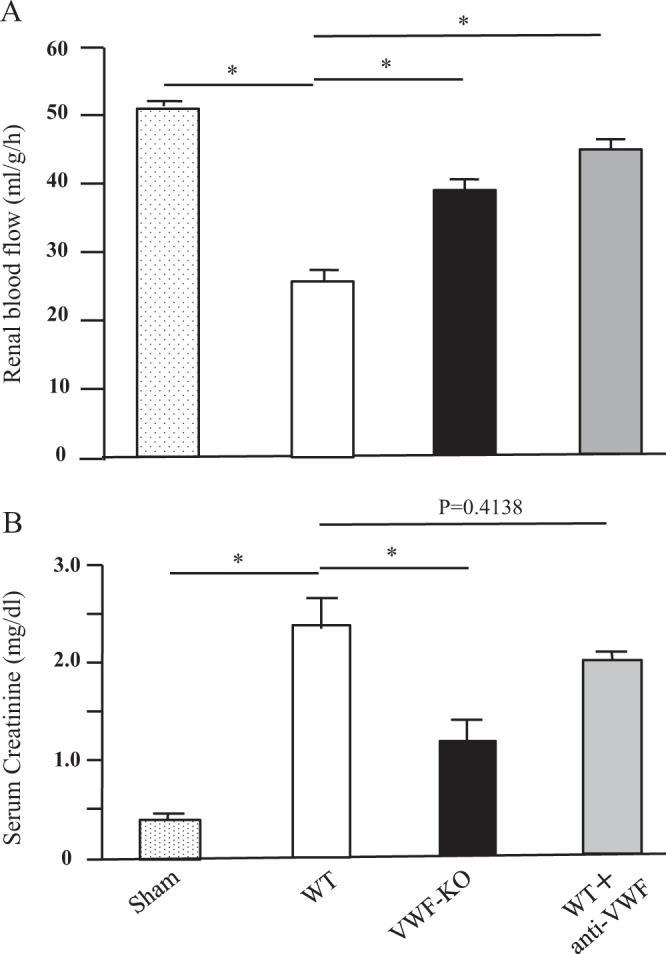
Effects of the function-blocking anti-VWF antibody NMC4 on RBF and serum creatinine in mice with I/R kidney injury. Experimental conditions were basically the same as those described in the Fig. 1 legend, except that the anti-VWF antibody NMC4 (10 μg/mouse) was injected intravenously instead of rADAMTS13. (**A**) Each bar indicates the mean actual RBF unit ± SEM (ml/g/h, n = 10, each group) at 24 h after reperfusion. Note that anti-VWF antibody significantly (*) improves the restoration of RBF in WT mice. (**B**) Bars indicate the mean ± SEM of serum creatinine values (n = 10, each group) at 24 h after reperfusion. The serum creatinine value in WT mice is also improved by antibody injection, although the difference is not statistically significant. These findings indicate the beneficial effects of the function-blocking anti-VWF antibody following mouse I/R kidney injury.

To further clarify the ameliorative effects of ADAMTS13 on I/R kidney injury, we studied another mechanism of VWF functional regulation using an anti-VWF antibody, NMC-4, that completely blocks VWF interaction with platelet GP Ib. NMC-4, similar to rADAMTS13, significantly improved RBF in WT mice at 24 h after reperfusion (from 25.8 ± 2.7 to 45.0 ± 1.9 ml/g/h, Fig. 4A). The serum creatinine value in WT mice was also improved by this antibody (from 2.40 ± 0.29 mg/dl to 2.11 ± 0.12 mg/dl, Fig. 4B), although this difference was not statistically significant.

## Discussion

Recent mouse model studies by our group and others demonstrated that VWF is critically involved in the pathophysiology of I/R injuries in various organs, such as the heart, brain and liver^12–15,17^. Thus, the present study was undertaken to address the question of whether VWF also plays a role in I/R kidney injury.

Our results revealed that following I/R kidney injury, VWF-KO mice exhibited less kidney damage than WT mice. Further, the functional regulation of VWF by ADAMTS13 or a function-blocking anti-VWF antibody significantly mitigated acute I/R damage in the mouse kidney (Fig. 4). Together, these results clearly indicated the critical involvement of VWF in the pathogenesis of I/R kidney injury.

Although the precise mechanisms by which VWF is involved in I/R injury have not yet been fully elucidated, ischemic damage to microvascular endothelial cells may play a crucial role. Injured endothelial cells release large amounts of ultra-large VWF multimers that are very active biologically, and then VWF-mediated platelet thrombus formation can proceed directly on the injured endothelial cell layers^6,7,18^. The resulting thrombotic occlusion of the microvasculature results in organ insufficiency, contributing at least in part to the pathophysiology of I/R injury. The effects of rADAMTS13 in our study suggest a crucial role of ultra-large VWF strings bound to stimulated endothelial cells. Interestingly, a recent study demonstrated the critical involvement of VWF-dependent intrarenal thrombosis in the progression of diabetic nephropathy in a mouse model of diabetes mellitus^19^. Thus, it is reasonable to assume that VWF-dependent platelet adhesion/aggregation in the renal microvasculature may play a role in the pathophysiology of I/R kidney injury, although no appreciable intravascular thrombosis was observed in the kidney tissue samples in the present histological study.

In addition to the thrombotic properties of VWF, VWF-mediated inflammatory responses may also be involved in the pathophysiology of I/R injury^20^. In this regard, several lines of evidence recently indicated that VWF plays a critical role in neutrophil recruitment during reperfusion; these cells can release free radicals and inflammatory factors such as interleukins at local ischemic sites^12,15,21,22^.

Our earlier studies^15,21^ revealed that the neutrophil infiltration at ischemic sites is crucially dependent upon VWF functions. Thus, we have focused on neutrophil recruitment in the present experimental approach, while other leukocytes may also contribute to the pathogenesis of the I/R injury. As a result, our histological study supported the idea that VWF is critically involved in neutrophil infiltration in kidney tissues following I/R injury (Fig. 3). Together with tissue damage caused by neutrophil-derived inflammatory cytokines, plugging of arterial capillaries by neutrophils can contribute to ischemic tissue damage in the reperfusion phase^15,23^. Leukocyte capillary plugging, as well as platelet micro-aggregates, are assumed to also contribute to microcirculatory insufficiency in I/R kidney injury^15,23,24^.

The precise mechanisms of VWF-mediated neutrophil recruitment remain to be clarified. However, our preceding study on hepatic I/R injury demonstrated the critical involvement of platelets in this regard^15^. In that study, our *in vivo* intravital imaging approach revealed that platelet interactions with both vessel walls and leukocytes were significantly reduced in VWF-KO mice. Thus, these platelet interactions, which can be mediated by VWF, play a key role in neutrophil recruitment in the reperfusion phase of I/R injury^15,18^. Alternatively, platelet interaction with VWF can support neutrophil interactions with activated endothelial cells, especially under rheological conditions with high shear stress such as those found in small arterioles or arterial capillaries, where VWF plays a paramount role in platelet function^6,7,18^. Indeed, this theory was supported by the effects of the function-blocking anti-VWF antibody in the present study (Fig. 4). Since this antibody completely blocks the interaction between VWF and platelet GP Ib, our results suggest the crucial involvement of VWF-platelet interaction in neutrophil recruitment in the present I/R paradigm.

In conclusion, VWF plays a role in the pathogenesis of AKI, in which both VWF-dependent thrombotic and inflammatory responses trigger tissue damage by thrombotic ischemia or inflammatory cytokines in the kidney. Our results are mostly compatible with a similar study by others^16^, which was recently published during the preparation of this manuscript, albeit with different experimental approaches. Thus, proper functional regulation of VWF is likely to improve the microcirculation and vessel functions in the kidney, suggesting a novel therapeutic potential against AKI. It is presently unknown whether the functional regulation of VWF in AKI can also carry over into improved chronic kidney injury or not. Further studies with long-term observation may address this question.

## Methods

### Mice

The present study was approved by the institutional review board of Nara Medical University and all methods were performed in accordance with the guidelines and regulations of the institution. Wild-type (WT) mice in C57BL/6 background were purchased from Japan SLC (Shizuoka, Japan) and VWF gene-deleted (VWF-KO) mice were obtained from the Jackson Laboratory (Bar Harbor, ME, USA), as previously described^15,21^. All mice used in this study were healthy males aged 8–12 weeks, and had body weights of 25–30 grams.

### Recombinant human ADAMTS13

The recombinant human ADAMTS13 (rADAMTS13) used in this study was previously described elsewhere^15,25,26^. In brief, rADAMTS13, previously designated as MDTCS, spans from the metalloproteinase (M) domain to the spacer (S) domain (amino acid residues 75–685) and possesses VWF-cleaving activity equivalent to that of the full-length ADAMTS13 molecule, as determined by the FRETS-VWF73 assay^15,27^. In some experiments, as indicated, rADAMTS13 (10 μg/mouse equivalent to 2800 U/kg) was injected intravenously in WT mice 15 min before the I/R operation.

### Anti-von Willebrand factor antibody NMC-4

The anti-human VWF monoclonal antibody NMC-4 used in this study was previously described in detail^28,29^. This antibody recognizes the A1 domain of VWF and can completely inhibit the VWF-platelet glycoprotein (GP) Ib interaction at a concentration of greater than 0.1 μg/ml. In some experiments, as indicated, NMC4 (10 μg/mouse) was injected intravenously in WT mice 15 min before the I/R operation.

### Mouse model of I/R kidney injury

All animal experiments were conducted with the permission of the Institutional Animal Care and Use Committee of Nara Medical University. In the present study, we employed hemi-renal mice, in which the right kidney was surgically removed by the standard mouse nephrectomy procedure 1 week prior to the I/R experiment. Our preliminary experiments indicated that neither significant increase of serum creatinine nor compensatory hypertrophy in the appearance of remaining kidney were detected by the hemi-nephrectomy procedure.

In brief, hemi-renal mice were placed in a prone position on a heating pad and anesthetized with continuously inhaled isoflurane (flow rate: 4.0 L/min for induction and 1.5 L/min for maintenance) administered using an isoflurane-specific vaporizer (MK-VAPO: Muromachi Kikai Co., Ltd, Tokyo, Japan) and anesthetizer (MK-A110: Muromachi Kikai Co., Ltd). Surgical incision was performed on the left side of the back and the left kidney was removed from the abdominal cavity for the duration of the operation. Both the renal artery and vein were clamped at the renal hilus using a clamping clip to induce 30-min ischemia. The RBF was monitored on the surface of renal hilus by Laser Doppler flowmetry (ALF21, Advance Co, Tokyo, Japan) with the attached probe (1 mm radius and output of 2 mW at the probe tip), as previously described^15^, and the actual RBF unit is expressed as a flow volume per unit kidney weight per unit time (ml/g/h). During the ischemic phase, the RBF was maintained at around 10% of the pre-ischemic value. The clip was then removed to provoke renal reperfusion, which was again confirmed by Laser flowmetry at 15 min after reperfusion. One hundred microliters of saline were injected into the peritoneal cavity before the final suture to prevent dehydration. The left kidney was then put back in the body and the skin incision was closed. As a negative control, some hemi-renal WT mice were sham-operated (open surgery without renal I/R). No mice demonstrated excessive blood loss during whole surgical procedure. The renal blood flow was measured again 24 h after reperfusion, and mice were sacrificed for blood collection and histological analysis of kidney tissue. Although a serum creatinine value is not an absolute evaluation of glomerular filtration, it could be useful for relative comparison between each group. Thus, we employed a serum creatinine value as an indicator for acute changes of renal functions in this study.

### Histological analysis of mouse kidneys

Extracted kidneys were fixed and stained with hematoxylin-eosin (HE) or Periodic Acid-Schiff (PAS), and renal tubular damages was evaluated using “tubular damage score” that was newly defined in this study. Using a computer-assisted image analyzer (Image J version:2.0.0-rc-43/1.50e), the tubular damage score was calculated based on the ratio of the area with loss of normal tubular construction (evaluated with HE samples) or with elimination of brush borders (evaluated with PAS samples) relative to the total area randomly selected on each tissue sample slide (microscopic frame: x400 magnification). The score was defined as follows: grade 1, 0 to 25% tubular necrotic area; grade 2, 26 to 50%; grade 3, 51 to 75%; and grade 4, greater than 75%.

In parallel, immunostaining with anti-VWF antibody (polyclonal rabbit antibody: DAKO. A0082, Agilent Pathology Solutions, Santa Clara, CA) was also employed to evaluate the VWF distribution in the area of renal tissue damages.

### Statistics

All data are described as means ± standard error (SEM) and were analyzed by one–way ANOVA (analysis of variance) and multiple comparison. P values < 0.05 were considered statistically significant.

### Ethical approval

All animal experiments were conducted with the permission of the Institutional Animal Care and Use Committee of Nara Medical University. This article does not contain any studies with human participants performed by any of the authors.

## Data Availability

All data generated or analysed during this study are included in this published article.
